# Enzyme Engineering Database (EnzEngDB): a platform for sharing and interpreting sequence–function relationships across protein engineering campaigns

**DOI:** 10.1093/nar/gkaf1142

**Published:** 2025-12-08

**Authors:** Yueming Long, Fatemeh Abbasinejad, Francesca-Zhoufan Li, Pierre Reinprecht, Bruce Wittmann, Jennifer L Kennemur, Hayden Carder, Jason Yang, Theophile Lambert, Ryen O’Meara, Lukas Radtke, Ziyang Qin, Sabine Brinkmann-Chen, Frances Arnold, Ariane Mora

**Affiliations:** Division of Chemistry and Chemical Engineering, California Institute of Technology, California Blvd., Pasadena, CA 91125, United States; Scientific Software Engineering Center, Whiting School of Engineering, Johns Hopkins University, 3400 N Charles St, Baltimore, MD 21218, United States; Division of Biology and Biological Engineering, California Institute of Technology, California Blvd., Pasadena, CA 91125, United States; Division of Chemistry and Chemical Engineering, California Institute of Technology, California Blvd., Pasadena, CA 91125, United States; Microsoft, Office of the Chief Scientific Officer, One Microsoft Way, Redmond, WA 98052, United States; Division of Chemistry and Chemical Engineering, California Institute of Technology, California Blvd., Pasadena, CA 91125, United States; Division of Chemistry and Chemical Engineering, California Institute of Technology, California Blvd., Pasadena, CA 91125, United States; Division of Chemistry and Chemical Engineering, California Institute of Technology, California Blvd., Pasadena, CA 91125, United States; Division of Chemistry and Chemical Engineering, California Institute of Technology, California Blvd., Pasadena, CA 91125, United States; Division of Chemistry and Chemical Engineering, California Institute of Technology, California Blvd., Pasadena, CA 91125, United States; Division of Chemistry and Chemical Engineering, California Institute of Technology, California Blvd., Pasadena, CA 91125, United States; Division of Chemistry and Chemical Engineering, California Institute of Technology, California Blvd., Pasadena, CA 91125, United States; Division of Chemistry and Chemical Engineering, California Institute of Technology, California Blvd., Pasadena, CA 91125, United States; Division of Chemistry and Chemical Engineering, California Institute of Technology, California Blvd., Pasadena, CA 91125, United States; Division of Biology and Biological Engineering, California Institute of Technology, California Blvd., Pasadena, CA 91125, United States; Division of Chemistry and Chemical Engineering, California Institute of Technology, California Blvd., Pasadena, CA 91125, United States; School of Chemistry and Molecular Biology, University of Queensland, Cooper Road, 4072, Queensland, Australia

## Abstract

The discovery and engineering of new enzymes is important across the bioeconomy, with diverse applications from foods to pharmaceuticals, sensors to agriculture. However, enzyme engineering, in particular machine learning-guided engineering, is hampered by a lack of data. Currently there exists no database designed to capture and interpret datasets created in this domain, nor are there easy analysis and visualisation tools. We developed the Enzyme Engineering Database to provide a centralized resource and an online analysis tool to consolidate sequence-function data from enzyme engineering campaigns, thereby making three contributions: (i) a database into which researchers can deposit public data, (ii) visualisation and analysis tools for protein engineers to analyse their own data or compare enzyme variants to other engineering campaigns, and (iii) a gold-standard dataset for benchmarking automated extraction along with the first large language model extraction pipeline specific for enzyme engineering campaigns. The Enzyme Engineering Database is accessible at http://enzengdb.org/.

## Graphical Abstract

**Graphical Abstract:**
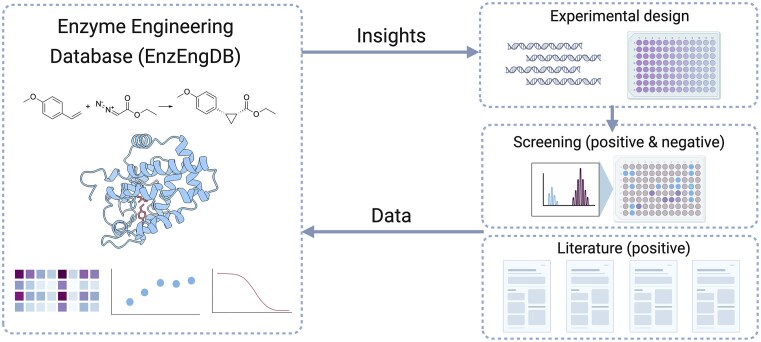

## Introduction

Enzymes are engineered using cycles of mutagenesis, or rational substitution, and selection or screening to enhance specific properties such as activity and selectivity [1–3]. Such engineering has enabled enzymes to catalyse non-natural reactions, such as carbene and nitrene transfers or reductive amination of non-native substrates, and to improve cold stability for laundry processing [4–6]. Enzyme engineering’s numerous techniques include directed evolution (DE) campaigns, targeted mutation studies, and machine learning (ML)-guided designs. Large, information-rich datasets emerge from the labour- and resource-intensive engineering workflows, and yet most data remain uninterpreted, unused, or unpublished due to the high cost of sequencing and lack of standardised analysis [7, 8]. While recent high-throughput workflows such as LevSeq, ParSeq, SequenceGenie, and others [9–12] have enabled the capture of plate-scale sequence-function maps during screening, these datasets stay siloed in individual labs, and researchers routinely juggle multiple scripts and spreadsheets to analyse one round of experiments before planning the next [13].

Current public databases partly address the need for an enzyme engineering data resource, but important gaps remain. Public resources such as UniProtKB [14], BRENDA [15], Rhea [16], and KEGG [17] consolidate enzyme information on primarily natural activities classified by enzyme commission (EC) numbers [18]. As a result, many engineered reactions and variants, such as *de novo* designed enzymes or repurposed enzymes that catalyse new-to-nature chemical reactions, are missing [19–22]. These databases are also highly curated and therefore contain mostly positive data, whereas ML models learn best from both positive and negative examples. ProtaBank [23] helps bridge this gap by accepting any mutational dataset; however, it is designed as a general-purpose deposition portal rather than an enzyme-specific resource with analysis features. Beyond experimental data, the scientific literature constitutes a rich yet non-standardised source of enzyme engineering data. Large language model (LLMs) and agentic systems offer practical routes to automated and standardised data collection from literature [24]. Recent pipelines such as Enzyme Co-Scientist [25] and EnzyExtract [26] have parsed thousands of preprint papers to extract kinetic constants (*k*_*cat*_, *K*_*M*_, *k*_*cat*_/*K*_*M*_), assembling >90 ,000 curated records with precision scores ranging from 0.8 to 0.9. However, these pipelines focus solely on kinetic constants and overlook sequence-function maps, negative variants, and activity metrics such as yield, turnover number (TTN), and selectivity, all of which are central metrics in enzyme engineering campaigns [27–29]. This underscores the need for a unified platform linking enzyme sequence, chemical transformation, and quantitative performance data, including both positive and negative results to accelerate discovery and improve reproducibility.

To address this gap, we developed the Enzyme Engineering Database (EnzEngDB), an open-source database coupled with an interactive web analysis tool. EnzEngDB is designed specifically for enzymes where both the catalyst and the chemical transformation are well defined: each record contains a canonical SMILES representation of the reaction (substrates to products), the full-length enzyme sequence, and quantitative performance metrics such as yield, turnover number (TTN), or selectivity. In its current release, EnzEngDB includes datasets spanning directed evolution campaigns, targeted mutation studies, and ML-guided designs, with examples from both natural and new-to-nature reactions. Our primary focus remains on transformations not found in nature, reflecting both the unique expertise of the founding laboratory and the scarcity of such data in existing resources, but the database structure is intentionally broad and designed to accommodate enzyme engineering datasets of all types. This design choice ensures compatibility with a wide range of present and future applications while giving immediate priority to the most information-rich, underrepresented datasets.

## Materials and methods

### Manual data collection and cleaning

For our gold standard dataset, we selected peer-reviewed enzyme engineering studies from our laboratory from the past decade and have included four additional combinatorial or model-guided mutation datasets that cover diverse enzyme engineering strategies. Working with these publications lets curators locate details quickly and cross-check against lab records, giving the most reliable baseline for benchmarking. The curators were three postdoctoral researchers, five PhD candidates, and two masters students. Curators independently extracted four fields from each paper: (i) enzyme/variant identifier, (ii) full-length amino-acid sequence, (iii) model reaction encoded as canonical SMILES (substrates to products), and (iv) performance values, SI 1. Notably, curators struggled to locate consistent sequence annotations and performance values because the data were scattered across the main text, figures, and supplementary files. Conflicts were resolved by a third curator, who consulted the original figures and deposited the consensus record. These manually extracted data ensured consistency between DNA and protein, parents and enzyme variants, and the validity of the SMILES using RDKit [30].

### Automated literature mining and data standardisation

**Figure 1.**
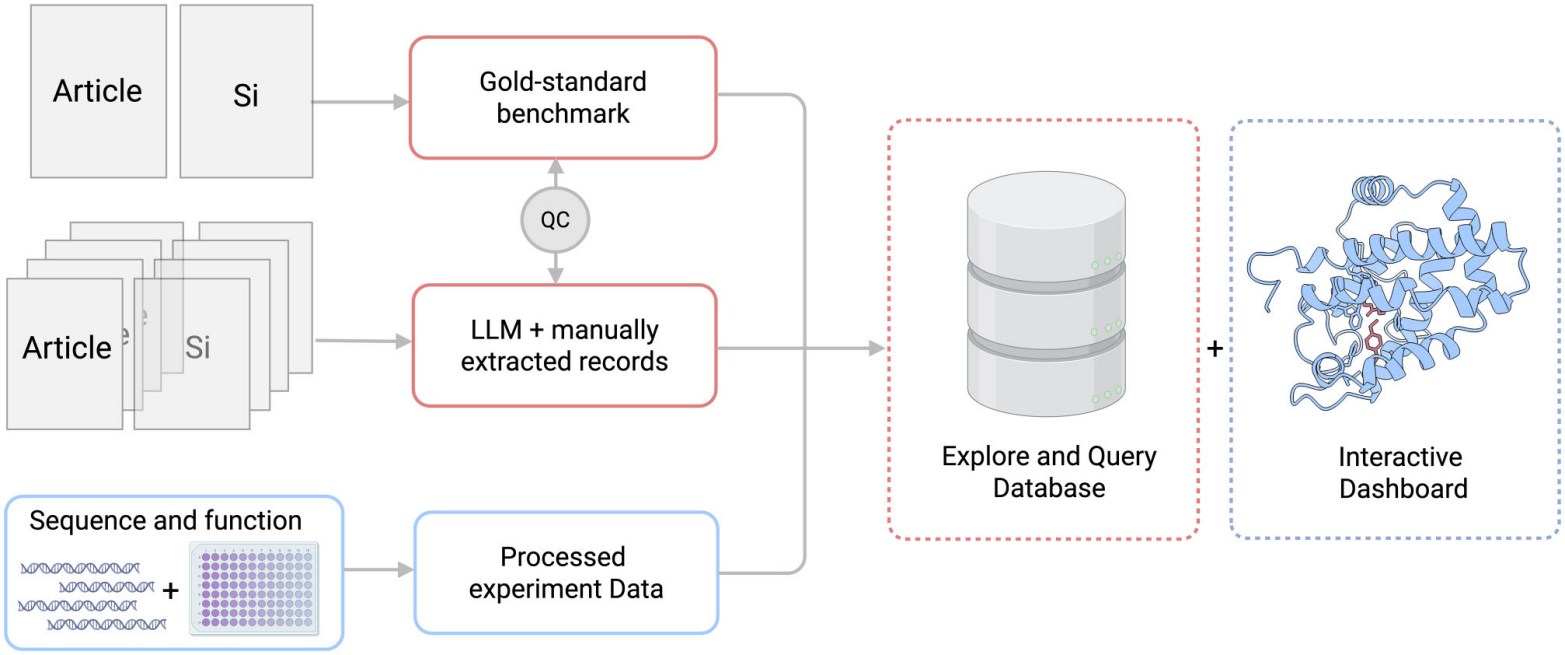
Integrated workflow for EnzEngDB population and visualisation. Literature PDFs are processed via two routes (i) manual curation to create a gold-standard benchmark and (ii) an LLM-based extraction pipeline that generates auto-extracted records. Both outputs, together with CSV uploads from plate-scale experiments, converge into a unified, schema-compliant CSV collection. These curated records feed an interactive front end that links sequence tables, fitness heat maps, and 3D structural views. Created in BioRender. Mora, A. (2026) https://BioRender.com/sirjd6g.

To scale curation beyond our manually compiled gold standard dataset, we built a four-step, LLM-based extraction pipeline, validated it on the gold standard papers, and then applied it to a wider literature set. We conducted a comprehensive PubMed search for papers related to enzyme engineering for unnatural chemistry to capture data that is not covered by existing resources, SI 2. This query retrieved 912 papers with DOIs. Abstracts were scored for relevance with the Gemini 2.5 Flash model, and those above 0.90 confidence yielded 327 candidate manuscripts, including 32 of our 34 gold-standard references; see SI 2 for download and scoring details. Full-text PDFs and supplements were downloaded from PubMed Central when possible. The pipeline identified engineered variants and their full-length sequences, sanitised the sequences, encoded each reported reaction as a canonical SMILES string, and pulled quantitative metrics such as yield or TTN into schema-compliant CSV and JSON files; see SI 3 for detailed information on the extraction method. Complete reaction, sequence, and performance records were recovered for 133 out of 327 papers, which were then manually validated; the rest lacked yield data or used chemical names the pipeline could not resolve. The same curators who prepared the gold standard set reviewed every output and ensured that the recorded sequences and reactions matched the source manuscripts (Fig. 1).

### Development of EnzEngDB

EnzEngDB was implemented in Python with the Plotly Dash web framework [https://plotly.com/dash] and packaged in a Docker container. All curated records and user-uploaded datasets are stored as CSV and .CIF files and are available for direct download on the website. Protein structures are displayed with Mol* through the Dash-Molstar wrapper [https://github.com/everburstSun/dash-molstar], and two-dimensional depictions of substrates and products are generated on the back end with RDKit. Sequence handling and alignment use BioPython (SI 3).

## Results

### Datasets consolidated in EnzEngDB

The present EnzEngDB release already ingests plate-scale CSV-formatted datasets produced by the LevSeq screening workflow. Additionally, any CSV file that conforms to the standard column headers specified in SI 1 can be uploaded to the database. A dedicated Upload tab in the database and an accompanying Information tab perform automatic checks for required columns before ingestion, ensuring standardisation and lowering the barrier to community participation. To demonstrate the platform’s capacity to store and analyse experimental results, we have deposited an error-prone polymerase chain reaction random mutagenesis dataset and a five-site combinatorial mutagenesis dataset, together representing sequence-function measurements equivalent to roughly eight 96-well plates of variants, each measured for two products.

**Figure 2.**
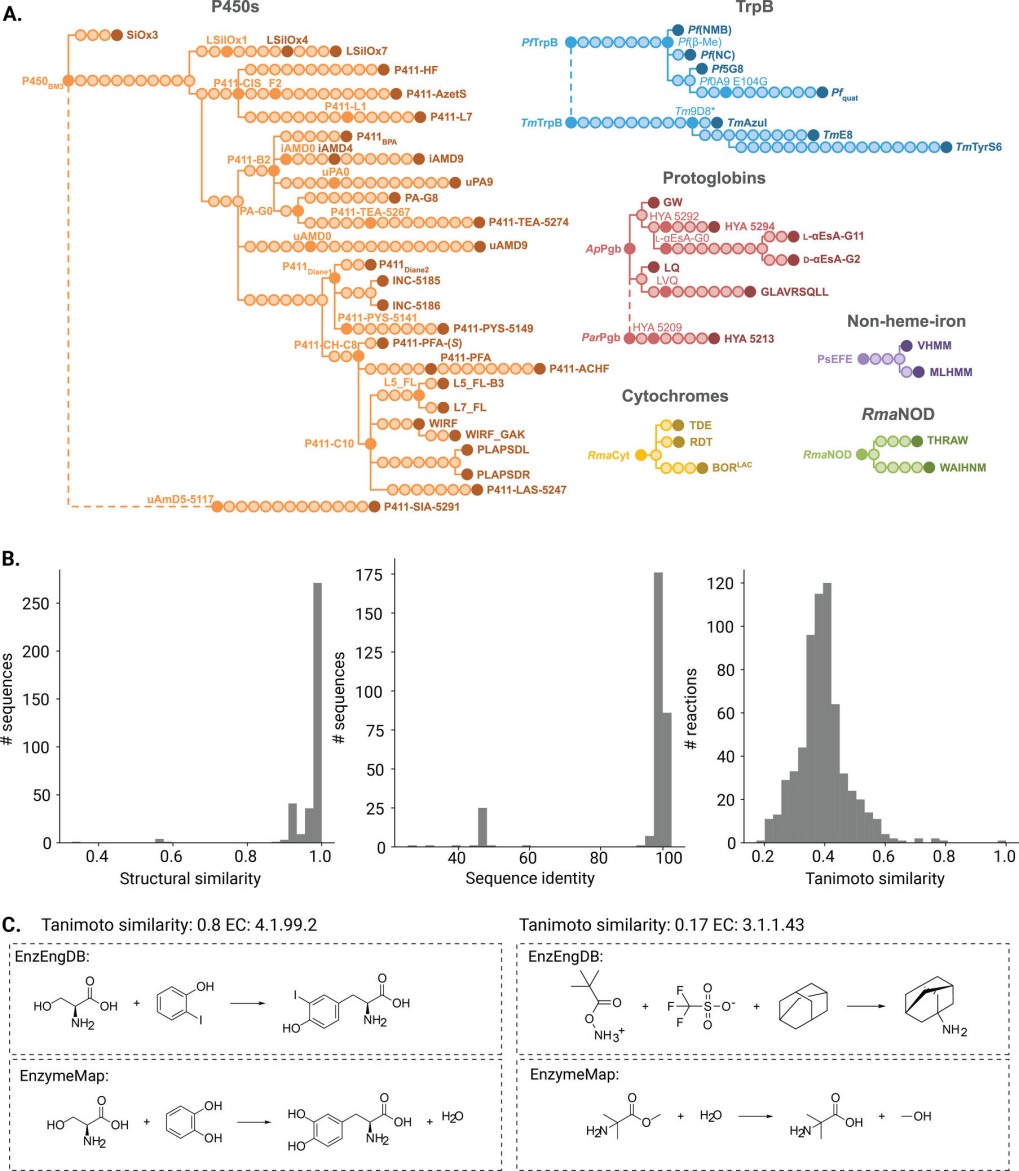
(**A**) Schematic representation of the six enzyme families across the 366 enzymes from directed evolution campaigns captured in the EnzEngDB manual gold-standard dataset (see SI 5 for publications). (**B**) The similarities between the gold standard section of the EnzEngDB and the publicly available databases are shown: structural similarity was compared to PDB, sequence identity to SwissProt, and the reactions were compared to unmapped reactions in EnzymeMap. (**C**) The most similar (that was not an exact match) and least similar reactions are shown alongside their matching reaction and EC classes in EnzymeMap to show the difference in the reactions between the EnzEngDB manual gold-standard dataset and EnzymeMap.

We also manually curated a gold-standard dataset comprising 6,234 sequence-function entries across 40 papers. The 635 reactions include carbene, nitrene, and condensation reactions, and the 1,846 unique protein variants include heme proteins and tryptophan synthase (Fig. 2A, Supplementary Fig. S4, and Supplementary Tables S1 and S2). These variants trace back to six protein scaffolds and span a wide range of non-natural substrates and transformations (Fig. 2A). Although their sequences show high similarity to entries in the PDB [31] and SwissProt, the chemistries they perform are distinct (Fig. 2B). Reaction space comparison, quantified with the Tanimoto similarity using RDKit structural fingerprints, indicates that most transformations in EnzEngDB are different from those in EnzymeMAP, confirming that EnzEngDB captures largely unique biocatalytic reactions (Fig. 2C). To complement the directed evolution studies, we include data from other enzyme engineering approaches such as combinatorial studies and ML-guided designs, including three non-natural substrate datasets (included above) and four native reaction datasets (447, 759 entries) (SI 4 and Supplementary Table S3).

As the manual extraction of an enzyme sequence and its corresponding function is time consuming, taking each curator approximately an hour per paper, we developed an automated extraction pipeline to expand our dataset. To evaluate the automated extraction pipeline, we reprocessed the PDFs and Supplementary files for every gold-standard paper, omitting those without sequence information. The extracted parent entries for each campaign were mapped to the reference set under following criteria: sequences could differ by terminal tag truncation, and reactions were deemed equivalent when the Tanimoto similarity exceeded 0.80 (SI 5). Under these criteria, 30% of the LLM extractions passed (SI 6). Papers from the automated extraction (133 downloaded papers) were manually validated and 64% required manual intervention for reasons such as no sequence or standard chemical name provided (SI 7). We found that the LLM extraction pipeline reduced the time to curate data, requiring correcting rather than searching the SI and PDF for information. Rerunning sequence extraction multiple times helps correct sequence-extraction errors, and providing full IUPAC names in the text reduces reaction-extraction errors. Common reaction extraction errors arise from missing stereochemistry information or a manual curator mistakenly extracting the wrong chemistry (SI 6). Moreover, our attempts to extract chemical structures directly from figures revealed that current LLMs and software packages still struggle to capture structural information directly. Common LLM sequence extraction errors were character issues (e.g. M as W); however, we also identified cases where an author deposited the incorrect sequence in the SI or referenced an incorrect PDB ID. The effort required to reconcile these details shows how easily critical information can be missed or misinterpreted and underscores the need for a community-wide, standard reporting format that records variant lineage, reaction context, and quantitative metrics at the time of publication. While automation reduced the average hands-on curation time from roughly an hour per paper to <10 min per paper, expert validation remains essential.

The extraction pipeline and manual validation produced an additional 2415 entries drawn from 102 papers, averaging 50 ,000 output tokens and ~50 cents per paper. These records cover 818 protein variants that catalyse 1,078 reactions.

### Interactive experiment dashboard

**Figure 3.**
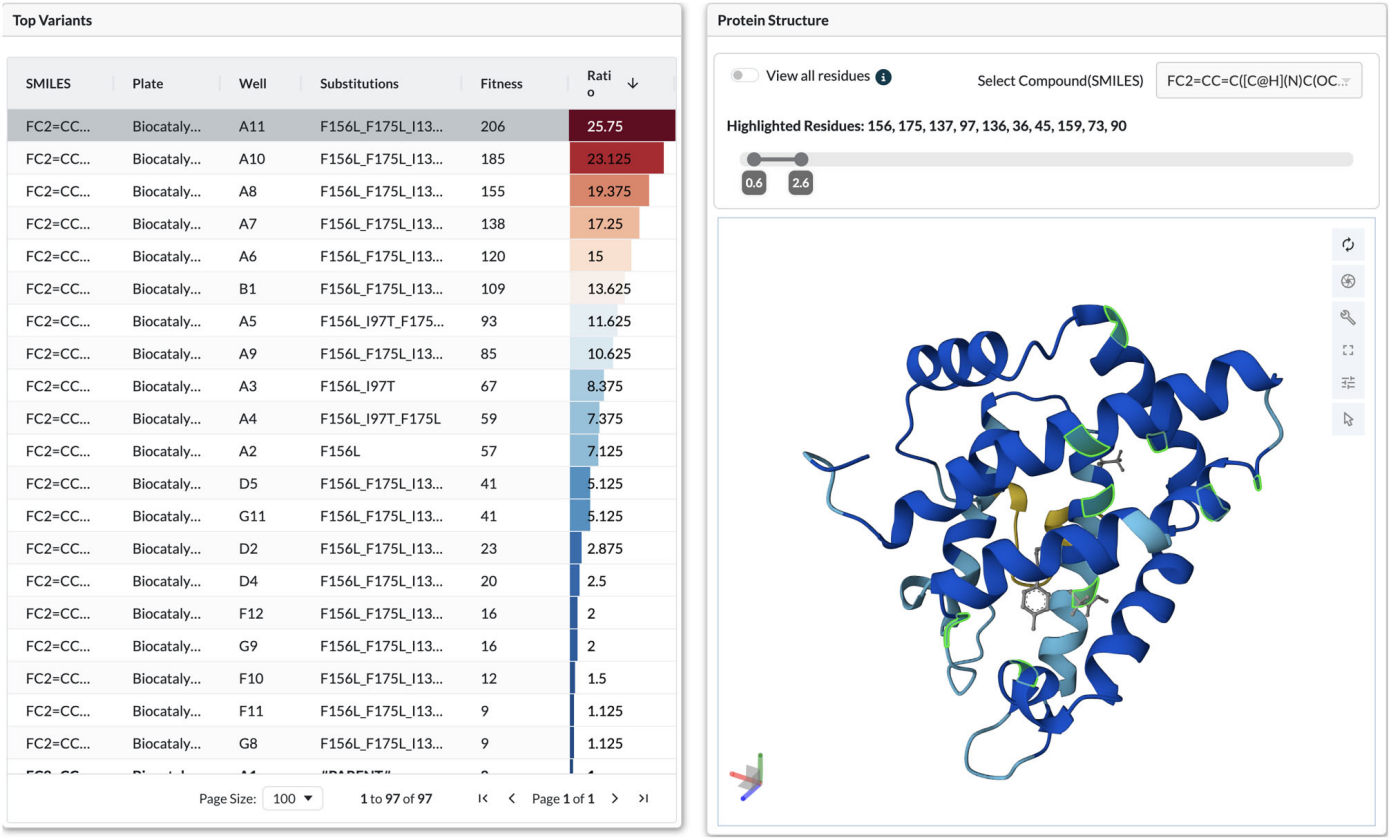
Interactive dashboard partial view: selected variant mutations (positions 156, 175, 137, 97, 136, 36, 45, 159, 73, and 90) highlighted on the 3D protein structure [37].

The EnzEngDB website includes over 10,000 experimentally collected records. For each enzyme engineering campaign an interactive dashboard collects all essential content in a single view: the parent amino acid sequence, experimental metadata, the canonical SMILES representation of the model reaction, and a manipulable protein structure (Fig. 3). The interactive three-dimensional protein structure is provided alongside a sortable table of variants listing each mutation, the parent-normalized fitness value, and the fold-change ratio. Clicking on a row highlights the mutated residues on the 3D model and auto-zooms to their local environment, letting users investigate structural effects from sequence substitutions that previously required separate scripts or multiple programmes (Fig. 3). The structure is rendered in a 3D viewer that supports zoom and rotation for in-depth analysis. Filters can be used to organise the variant list by any metric, and the protein viewer will remain synchronized with selected row. The integrations of a protein viewer and spreadsheet-style analytics turn raw screening data into an exploratory workspace, reducing the time from data upload to actionable hypotheses.

### Database browsing and queries

**Figure 4.**
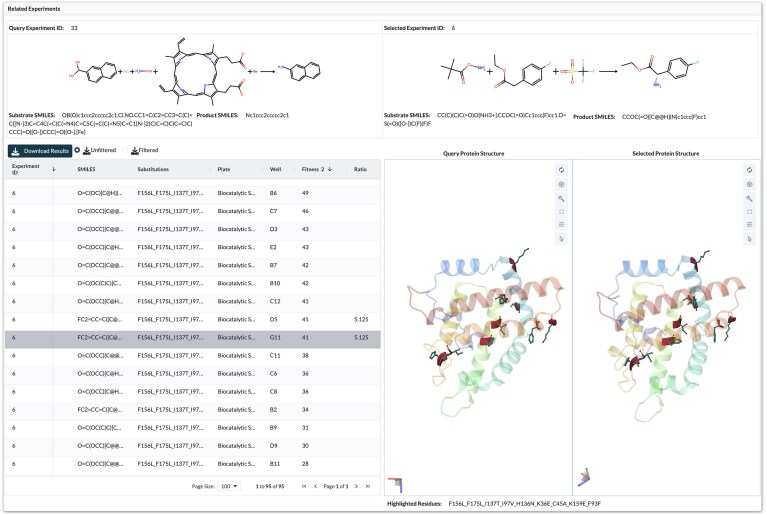
An example of a sequence-similarity search within the protoglobin nitrene-transfer lineage reveals an active-site mutation that enhances selectivity yet remains unexplored in other campaigns [37, 38].

All campaigns in EnzEngDB can be queried and compared using the web interface. While the visualisation dashboard is optimized for directed evolution datasets, where substitutions are highlighted on the protein structure and variant fitness can be compared to a parent, the database can also accommodate other enzyme engineering campaigns, provided each entry includes a reaction SMILES and a quantitative product measurement. From the dashboard, users can run a sequence similarity search to find enzymes with different functions or to identify mutations that improve function in other campaigns, with results filterable by experiment name, percentage sequence identity, reaction SMILES, or assay type. For every dataset with a specified parent, variant fitness is automatically reported as a fold change relative to that reference, while the others are reported as the extracted values. These normalized values reveal gain- and loss-of-function mutations and can guide the choice of target sites for the next library. For instance, a similarity search on the protoglobin nitrene-transfer lineage reveals an active-site substitution that enhances selectivity but has yet to be explored in other protoglobin projects, flagging a promising engineering target without relying on informal knowledge transfer (Fig. 4).

### Private and public instances of EnzEngDB

To incentivize participation, the database includes both a private and a public instance. The private instance provides full visualisation and formatting functions to help researchers store and analyse their own data locally, while the public instance is intended for deposition once datasets are published. The private instance can be locally served using one line of code deploying a Docker container, so the data is stored and standardised for local use. Once the owner makes the data public, the public database instance will ingest the standardised screening data. Every dataset in the public instance can include a DOI link, a unique ID, and a downloadable BibTeX file during download to facilitate proper attribution and citation for data usage.

## Discussion

We developed the EnzEngDB as a data deposition hub that incentivizes researchers to share their screening data by providing interactive visualisations, enabling faster insights into gain-of-function mutations, and reducing the analysis workload. In addition to the public repository, EnzEngDB includes a private instance that allows researchers to store, format, and visualise their own data locally prior to publication. Public datasets with assigned DOIs will be accompanied by BibTeX files to ensure appropriate attribution to the original authors.

The framework of EnzEngDB is organised around amino acid sequence, fitness value, and fitness type, allowing a wide range of assay outputs to be captured in a consistent format. Whether the data originate from kinetic measurements (e.g. *K*_*M*_ or *k*_*cat*_), spectroscopic readouts, or other experimental assays, the database structure allows data to be stored, filtered, and processed in a consistent manner by representing them as fitness values. While not yet comprehensive, EnzEngDB should be regarded as an ongoing effort, with its scope expected to broaden as additional datasets are contributed and standardized ingestion pipelines are developed. Direct comparisons are recommended only within individual CSVs, where experimental conditions and formats are consistent. This design choice enables flexibility across diverse enzyme engineering campaigns while maintaining internal consistency.

We envision that EnzEngDB will convert siloed datasets into accessible resources, improving reproducibility, data access, and the creation of balanced datasets by including negative results. While these values may differ across campaigns, they are measured under consistent conditions within each dataset and are included in the downloadable CSV. For DE campaigns with a defined lineage, identical reaction conditions allow fold-change calculations, enabling standardised comparisons of mutation effects. We envision that this data will support the design of ML models that can be used to guide engineering, reducing the experimental burden [32–34]. Ultimately, systematic data collection of diverse enzyme engineering campaigns will bridge the gap between resource-intensive experiments and efficient, data-driven engineering methods [35, 36].

Alongside the platform, we assembled a manually curated set of new-to-nature reactions and used it to benchmark an LLM-based extraction pipeline, establishing the first benchmark for future automated curation. While the present EnzEngDB version shows that enzyme engineering data can be consolidated and searched in one place, coverage remains limited. The curated dataset mostly comes from a single laboratory, and every automatically extracted paper required expert validation, which is evidence that LLM extraction alone cannot yet capture the full detail of directed evolution campaigns and underscores the value of manual curation by domain experts.

Community participation is essential for EnzEngDB to become the comprehensive, standardised resource the field needs. As the field increasingly moves towards ML applications, it is essential to ensure that the extensive experimental effort required to generate training data is appropriately acknowledged. Even relatively modest ML tasks, such as fine-tuning, can demand substantial volumes of high-quality measurements. EnzEngDB was designed in part to address this need by providing clear mechanisms for dataset sharing and attribution for experimentalists who upload data.

We encourage researchers to deposit complete datasets in the format compatible with EnzEngDB, including both positive and negative data, at the point of screening and publication, and we invite developers to test and improve extraction scripts against the benchmark set. EnzEngDB is currently compatible with LevSeq. However, we make our code open source and encourage community-developed integrations with other sequencing methods via GitHub (https://github.com/ssec-jhu/levseq-dash). These contributions will turn EnzEngDB into a resource that can advance the community’s understanding of biocatalysis and provide new methods in data-driven enzyme engineering and design.

## Supplementary Material

gkaf1142_Supplemental_Files

## Data Availability

Enzyme Engineering Database (EnzEngDB) is available at http://enzengdb.org/. For analyses and LLM pipeline, see https://github.com/fhalab/EnzymeEngineeringDB and https://doi.org/10.5281/zenodo.17310823.

## Notes

Present address: NBC and Environmental Protection Technology Division, Armaments and Defence Technology Agency, 1090 Vienna, Austria

Present address: Université Paris-Saclay, CNRS UMR8182, Institut de Chimie Moléculaire et des Matériaux d’Orsay (ICMMO), 17 Avenue des Sciences, 91400 Orsay, France

Present address: AITHYRA GmbH, Research Institute for Biomedical Artificial Intelligence of the Austrian Academy of Sciences, Helmut-Qualtinger-Gasse 2, Stg. 2, A-1030 Vienna, Austria

Present address: Department of Biosystems Science and Engineering, ETH Zurich, Schanzenstrasse 44, Basel, 4056, Switzerland
