# Supplementary material for: Enzyme Engineering Database (EnzEngDB): a platform for sharing and interpreting sequence–function relationships across protein engineering campaigns
Source: Nucleic Acids Res. 2025 Dec 8;54(D1):D564–71. doi: 10.1093/nar/gkaf1142 (PMC12807667; doi:10.1093/nar/gkaf1142)
Supplement: gkaf1142_Supplemental_Files [file gkaf1142_supplemental_files.zip › EnzEngDB_SI_20250922.pdf]

SUPPLEMENTARY DATA

SI 1: **Data format and Dashboard Detail**

The final CSV contains one row per variant per reaction with columns in Figure S1. There are 15 columns in total.

Four columns are essential for retaining all information about an enzymatic reaction: aa_sequence, reaction_smiles, fitness_value and additional_information.

- The **aa_sequence** column stores the cleaned full-length amino-acid string
- The **reaction_smiles** provides a machine-readable representation of the model reaction
- The **fitness_value** reports the activity metrics
- The **additional_information** preserves all other assay parameters.

Additional columns required for upload: id, plate, well, amino_acid_substitutions. These are essential for visualization and direct ingestion by DEDB while retaining context for downstream analysis.

Extra columns are information retained during the extraction pipeline and will be stored in addition to the essential columns and the user can download the full CSV with all information.

id	barcode_plate	plate	well	smiles_string	smiles_reaction	alignment_count	alignment_probability	nucleotide_mutation	amino_acid_substitutions	nt_sequence	aa_sequence	fitness_value	fitness_type	additional_information
sL407C-1	1	Plate_1	A01	CCCCCCCCC	O=C(O)CCCCCCC	1	1				TIKEMPQPKTFG	230	ttn	{"x_axis_label":"sL407C-1","
dF393W-	1	Plate_1	A02	CCCCCCCCC	O=C(O)CCCCCCC	1	1		F393W		TIKEMPQPKTFG	192	ttn	{"x_axis_label":"dF393W-1",
dQ403W-	1	Plate_1	A03	CCCCCCCCC	O=C(O)CCCCCCC	1	1		Q403W		TIKEMPQPKTFG	420	ttn	{"x_axis_label":"dQ403W-1"

Figure S1. Final output CSV format compatible with the Enzyme Engineering Database upload.

The visualization dashboard has the following details, rows in the table include a fold-change column, color-coded red for gain-of-function and blue for loss-of-function, and bidirectionally linked to the structural model. Selecting a variant highlights the mutated residues in the model and automatically zooms to the affected region. For campaigns with plate-scale data, the dashboard displays a 96-well grid heat map, where each well is shaded by activity and annotated with its corresponding mutation string. A retention-of-function curve is plotted alongside the heat map, enabling rapid assessment of library quality and mutation effects. Users can sort or filter the variant table, choose which activity metric populates the heat map, switch between plates when multiple plates are available, and hover over any cell to reveal variant details. All actions dynamically update both the colored heat map and the 3-D viewer, highlighting the corresponding residues and zooming to their local environment.

SI 2: **PubMed Search Query**

The search query was performed on July 1, 2025. A set of keywords for directed evolution and a list of relevant authors were manually identified, and the specific query is shown below.

```
"""( ( "enzyme engineering"[tiab] OR "engineered enzyme"[tiab] OR "protein engineering"[tiab] OR "directed evolution"[tiab] OR "site-directed mutagenesis"[tiab] OR "enzyme design"[tiab] OR "designer enzyme"[tiab] OR "enzyme reprogramming"[tiab] OR "artificial enzym*"[tiab] OR "artificial metalloenzyme"[tiab] OR "de novo enzyme"[tiab] OR "synthetic enzyme"[tiab] OR "Hemeproteins"[mh] OR "Protein Engineering"[mh] OR "Biocatalysis"[mh] OR ( ("novel"[tiab] OR "new"[tiab]) AND ( "halogenase*"[tiab] OR "protoglobin*"[tiab] OR "cytochrome P450"[tiab] OR "P450"[tiab] OR "CYP"[tiab] OR "peroxidase*"[tiab] OR "monooxygenase*"[tiab] OR "transaminase*"[tiab] OR "aminotransferase*"[tiab] OR "hydrolase*"[tiab] OR "esterase*"[tiab] OR "lipase*"[tiab] OR "lyase*"[tiab] OR "aldolase*"[tiab] OR "oxidoreductase*"[tiab] OR "dehydrogenase*"[tiab] OR "metalloenzyme*"[tiab] OR "carbene"[tiab] OR "nitrene"[tiab] ) ) ) AND ( "non-natural"[tiab] OR "unnatural"[tiab] OR "abiotic"[tiab] OR "new-to-nature"[tiab] OR "non-native"[tiab] OR "xenobiotic"[tiab] OR "abiological"[tiab] OR "noncanonical"[tiab] ) AND ( "reaction"[tiab] OR "catalysis"[tiab] OR "chemistry"[tiab] OR "transformation"[tiab] ) ) OR ( ( "Arnold FH"[lastau] OR "Reetz MT"[lastau] OR "Fasan R"[lastau] OR "Zhao H"[lastau] OR "Baker D"[lastau] OR "Roelfes G"[lastau] OR "Ward TR"[lastau] OR "Lu Y"[lastau] OR "Hyster TA"[lastau] OR "Liu Z"[lastau] OR "Lewis JC"[lastau] OR "Coelho PS"[lastau] OR "Buller AR"[lastau] OR "Hilvert D"[lastau] OR "Kast P"[lastau] OR "Garcia-Borràs M"[lastau] ) AND ( ("engineering"[tiab] AND "halogenase"[tiab]) OR ("designer"[tiab] AND "enzyme"[tiab]) OR "enzyme engineering"[tiab] OR ("artificial"[tiab] AND "enzym*"[tiab]) OR ("artificial"[tiab] AND "metalloenzym*"[tiab]) OR "directed evolution"[tiab] OR ("non-native"[tiab] AND "reaction"[tiab] AND "enzym*"[tiab]) OR ("abiological"[tiab] AND "catalys*"[tiab] AND "enzym*"[tiab]) OR ("non-natural"[tiab] AND "reacti*"[tiab] AND "enzym*"[tiab]) OR ("noncanonical"[tiab] AND "activity"[tiab] AND "enzym*"[tiab]) OR ("novel"[tiab] AND "chemistr*"[tiab] AND "enzym*"[tiab]) OR "artificial enzym*"[tiab] OR "artificial metalloenzym*"[tiab] OR "engineered enzyme*"[tiab] OR "site-directed mutagenesis"[tiab] OR "enzyme design"[tiab] OR "designer enzyme*"[tiab] OR "enzyme reprogramming"[tiab] OR "de novo enzyme*"[tiab] OR "synthetic enzyme*"[tiab] OR "hemeprotein*"[mh] OR "protein engineering"[mh] OR "biocatalysis"[mh] OR "enzymatic platform"[tiab] OR "biocatal*"[tiab] OR "biocatalyst"[tiab] OR "biocatalytic platform"[tiab] OR "enzymatic synthesis"[tiab] OR "enzymatic reaction*"[tiab] OR "enzymatic transformation*"[tiab] OR "enzyme cataly*"[tiab] OR "enzymatic assembly"[tiab] OR ("enzymatic"[tiab] AND "insertion"[tiab]) OR "cytochrome P450*"[tiab] OR "P450*"[tiab] OR "CYP"[tiab] OR "P411*"[tiab] OR "peroxidase*"[tiab] OR "monooxygenase*"[tiab] OR "transaminase*"[tiab] OR "aminotransferase*"[tiab] OR "hydrolase*"[tiab] OR "esterase*"[tiab] OR "lipase*"[tiab] ) ) AND ( "biocatal*"[tiab] OR "catalytic activity"[tiab] OR "enzym*"[tiab] ) AND english[Language] AND ("2015/01/01"[PDAT] : "2025/12/31"[PDAT]) NOT (review[Publication Type] OR preprint[Publication Type] OR editorial[Publication Type]) """
```

SI 3: **Literature Retrieval and LLM Data Extraction**

Using the search query above, we compiled a set of relevant publications and then asked Gemini-2.5-Flash to classify each manuscript as either relevant or irrelevant to our database, returning a confidence score based on the title and abstract. The abstract classification prompt is shown below.

Gemini-2.5-Flash prompt for abstract classification:

```
"""
You are a scientific literature reviewer. Your task is to determine if a research paper is relevant to specific requirements.
REQUIREMENTS:
I want to extract the following information from the scientific literature:
Sequences and functions of enzymes that perform new-to-nature chemistry.
Therefore, I am searching for papers that report enzyme engineering research aimed at enabling new-to-nature transformations.
The papers must include the amino acid sequence (or at least sequence IDs) and functional data of enzymes (e.g., substrate, product, yield, enantioselectivity [ee], total turnover number [TTN], etc.).
The following are considered irrelevant:
- Purely computational studies (unless the predictions are experimentally confirmed)
- Binding proteins or non-catalytic proteins — only enzymes with catalytic activity are relevant
- Method papers without experimentally validated enzyme examples
- Review articles (as they do not contain novel information)
- Strain engineering or catalytic cascade studies unless data on individual enzymes is provided

PAPER TITLE: {title}
ABSTRACT: {abstract}
Analyze if this paper is directly relevant to the requirements. Consider:
- Does the paper address the main concepts of the requirements?
- Are the research methods, findings, or conclusions related to the requirements?
- Would this paper be useful for someone researching the requirements?
Respond in the following JSON format:

{{
        "is_relevant": boolean,
        "confidence": 0.0-1.0,
        "reasoning": "Brief explanation of your decision"
}}
Be strict in your evaluation - only mark as relevant if there's a clear connection to the requirements.
"""
```

For any paper with a confidence score above 0.9, we manually downloaded the manuscript and SI in PDF format. The two PDFs were then processed through the API-based automatic sequence–fitness CSV generation pipeline. (https://github.com/YuemingLong/DEBase/)

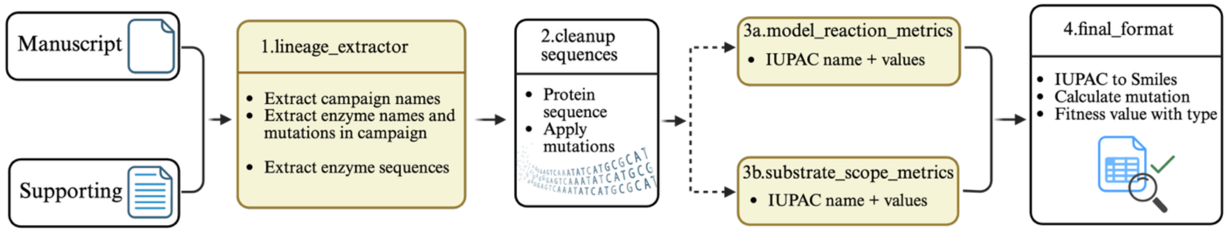

Figure S2. Pipeline for LLM based extraction.

We divided the generation of the sequence-fitness CSV into five steps, each guided by specific prompts to reduce context drift in the language model.

First, the code instructs Gemini-2.5-Flash to count the engineering campaigns reported in the paper; each campaign ID then serves as a primary key for retrieving every enzyme name and its mutations from the single table containing the complete mutation list.

Second, these enzyme names serve as a secondary key for extracting their DNA sequences. The prompt directs the model to copy each sequence exactly as printed. Because DNA strings are repetitive and error-prone, all spaces are removed and any sequence containing a stop codon before the terminus is discarded.

Third, the remaining DNA is cleaned and translated into protein. The listed mutations are applied only when the wild-type residue at the target position matches; otherwise, the sequence cell is left blank and flagged for manual review. This yields a complete sequence CSV, which is then passed to the model reaction extractor to gather substrate and product identifiers, IUPAC names, and performance metrics, prioritizing the table containing the full dataset.

Fourth, the substrate scope extractor runs with similar logic, but its prompt excludes the model reaction and asks Gemini to state why each entry lies outside that scope.

Finally, a cleanup script converts all IUPAC names to canonical SMILES via NCI, OPSIN, and PubMed, recalculates amino acid changes against the first variant of each campaign, reverse-translates DNA substitutions, and appends both the fitness value and its type to the finished output.

SI 4: **Data types and sources in version 1 of EnzEngDB**

S. Table 1. Manually and LLM curated datasets

Paper Title	source	doi
An Enzymatic Platform for Primary Amination of 1-Aryl-2-alkyl Alkynes	In-house	10.1021/jacs.1c11340
An enzymatic platform for the asymmetric amination of primary, secondary and tertiary C(sp^3)-H bonds	In-house	10.1038/s41557-019-0343-5
Asymmetric Alkylation of Ketones Catalyzed by Engineered TrpB	In-house	10.1002/anie.202106938
Biocatalytic Carbene Transfer Using Diazirines	In-house	10.1021/jacs.2c02723
Biocatalytic Construction of Chiral Pyrrolidines and Indolines via Intramolecular C(sp^3)-H Amination	In-house	10.1021/acscentsci.3c00516
Biocatalytic One-Carbon Ring Expansion of Aziridines to Azetidines via a Highly Enantioselective [1,2]-Stevens Rearrangement	In-house	10.1021/jacs.2c00251
Biocatalytic Synthesis of α-Amino Esters via Nitrene C-H Insertion	In-house	10.1021/jacs.4c09989
Biocatalytic, Intermolecular C-H Bond Functionalization for the Synthesis of Enantioenriched Amides	In-house	10.1002/anie.202110873
Biocatalytic, Stereoconvergent Alkylation of (Z/E)-Trisubstituted Silyl Enol Ethers	In-house	10.1038/s44160-023-00431-2
Biocatalytic, Enantioenriched Primary Amination of Tertiary C-H Bonds	In-house	10.1038/s41929-024-01149-w
Chemodivergent C(sp^3)-H and C(sp^2)-H Cyanomethylation Using Engineered Carbene Transferases	In-house	10.1038/s41929-022-00908-x
Direct Enzymatic Synthesis of a Deep-Blue Fluorescent Noncanonical Amino Acid from Azulene and Serine	In-house	10.1002/cbic.201900497
Directed Evolution of a Cytochrome P450 Carbene Transferase for Selective Functionalization of Cyclic Compounds	In-house	10.1021/jacs.9b02931
Directed evolution of cytochrome c for carbon-silicon bond formation: Bringing silicon to life	In-house	10.1126/science.aah6219
Directed evolution of enzymatic silicon-carbon bond cleavage in siloxanes	In-house	10.1126/science.adi5554
Diversity-Oriented Enzymatic Synthesis of Cyclopropane Building Blocks	In-house	10.1021/acscatal.0c01888

Dual-Function Enzyme Catalysis for Enantioselective Carbon-Nitrogen Bond Formation	In-house	10.1038/s41557-021-00794-z
Enantio- and Diastereoenriched Enzymatic Synthesis of 1,2,3-Polysubstituted Cyclopropanes from (Z/ E)-Trisubstituted Enol Acetates	In-house	10.1021/jacs.3c04870
Enantiodivergent α-Amino C-H Fluoroalkylation Catalyzed by Engineered Cytochrome P450s	In-house	10.1021/jacs.9b04344
Engineered Cytochrome c-Catalyzed Lactone-Carbene B-H Insertion	In-house	10.1055/s-0037-1611662
Engineering Cytochrome P450s for Enantioselective Cyclopropenation of Internal Alkynes	In-house	10.1021/jacs.0c01313
Enzymatic Assembly of Diverse Lactone Structures: An Intramolecular C-H Functionalization Strategy	In-house	10.1021/jacs.3c11722
Enzymatic Lactone-Carbene C–H Insertion to Build Contiguous Chiral Centers	In-house	10.1021/acscatal.0c01349
Enzymatic Nitrogen Incorporation Using Hydroxylamine	In-house	10.1021/jacs.3c08053
Enzymatic Nitrogen Insertion into Unactivated C-H Bonds	In-house	10.1021/jacs.2c08285
Enzymatic Primary Amination of Benzylic and Allylic C(sp^3)-H Bonds	In-house	10.1021/jacs.0c03428
Expanding Biocatalysis for Organosilane Functionalization: Enantioselective Nitrene Transfer to Benzylic Si–C–H Bonds	In-house	10.1021/acscatal.3c05370
Genetically programmed chiral organoborane synthesis	In-house	10.1038/nature24996
Nitrene Transfer Catalyzed by a Non-Heme Iron Enzyme and Enhanced by Non-Native Small-Molecule Ligands	In-house	10.1021/jacs.9b11608
Nitroalkanes as Versatile Nucleophiles for Enzymatic Synthesis of Noncanonical Amino Acids	In-house	10.1021/acscatal.9b02089
Protoglobin-Catalyzed Formation of cis-Trifluoromethyl-Substituted Cyclopropanes by Carbene Transfer	In-house	10.1002/anie.202208936
Reversing the Enantioselectivity of Enzymatic Carbene N-H Insertion Through Mechanism-Guided Protein Engineering	In-house	10.1002/anie.202303879
Selective Enzymatic Oxidation of Silanes to Silanols	In-house	10.1002/anie.202002861
Stereospecific Enzymatic Conversion of Boronic Acids to Amines	In-house	10.1021/jacs.4c04190
Tailoring Tryptophan Synthase TrpB for Selective Quaternary Carbon Bond Formation	In-house	10.1021/jacs.9b09864

The β-subunit of tryptophan synthase is a latent tyrosine synthase	In-house	10.1038/s41589-024-01619-z
A cell-penetrating artificial metalloenzyme regulates a gene switch in a designer mammalian cell	LLM	10.1038/s41467-018-04440-0
A designer enzyme for hydrazone and oxime formation featuring an unnatural catalytic aniline residue	LLM	10.1038/s41557-018-0082-z
A Diverse Library of Chiral Cyclopropane Scaffolds via Chemoenzymatic Assembly and Diversification of Cyclopropyl Ketones	LLM	10.1021/jacs.0c09504
A Photoenzymatic Strategy for Radical-Mediated Stereoselective Hydroalkylation with Diazo Compounds	LLM	10.1002/anie.202214135
Accessing non-natural reactivity by irradiating nicotinamide-dependent enzymes with light	LLM	10.1038/nature20569
An Artificial Heme Enzyme for Cyclopropanation Reactions	LLM	10.1002/anie.201802946
An Enzymatic Platform for Aniline Synthesis Through Oxidative Amination	LLM	10.1002/anie.202505252
An Enzymatic Platform for Asymmetric Synthesis of Si-Stereogenic Silanols	LLM	10.1002/anie.202501524
An Enzymatic Platform for the Highly Enantioselective and Stereodivergent Construction of Cyclopropyl-δ-lactones	LLM	10.1002/anie.202007953
Biocatalytic asymmetric aldol addition into unactivated ketones	LLM	10.1038/s41557-024-01647-1
Biocatalytic Strategy for Highly Diastereo- and Enantioselective Synthesis of 2 3-Dihydrobenzofuran-Based Tricyclic Scaffolds	LLM	10.1002/anie.201903455
Biocatalytic Strategy for the Highly Stereoselective Synthesis of CHF sub 2 sub -Containing Trisubstituted Cyclopropanes	LLM	10.1002/anie.202015895
Biocatalytic Synthesis of Allylic and Allenyl Sulfides through a Myoglobin-Catalyzed Doyle-Kirmse Reaction	LLM	10.1002/anie.201607278
Catalytic iron-carbene intermediate revealed in a cytochrome c carbene transferase	LLM	10.1073/pnas.1807027115
Catalytic promiscuity enabled by photoredox catalysis in nicotinamide-dependent oxidoreductases	LLM	10.1038/s41557-018-0059-y

Chemoselective Cyclopropanation over Carbene Y-H Insertion Catalyzed by an Engineered Carbene Transferase	LLM	10.1021/acs.joc.8b00946
Dehaloperoxidase Catalyzed Stereoselective Synthesis of Cyclopropanol Esters	LLM	10.1021/acs.joc.2c02030
Design and Evolution of an Artificial Friedel-Crafts Alkylation Enzyme Featuring an Organoboronic Acid Residue	LLM	10.1021/jacs.4c03795
Design and Evolution of an Enzyme for the Asymmetric Michael Addition of Cyclic Ketones to Nitroolefins by Enamine Catalysis	LLM	10.1002/anie.202404312
Directed Evolution of a Fluorinase for Improved Fluorination Efficiency with a Non-native Substrate	LLM	10.1002/anie.201606722
Directed Evolution of an Artificial Imine Reductase	LLM	10.1002/anie.201711016
Diverse Engineered Heme Proteins Enable Stereodivergent Cyclopropanation of Unactivated Alkenes	LLM	10.1021/acscentsci.7b00548
A De Novo Metalloenzyme for Cerium Photoredox Catalysis	LLM	10.1021/jacs.4c04618
Abiotic reduction of ketones with silanes catalysed by carbonic anhydrase through an enzymatic zinc hydride	LLM	10.1038/s41557-020-00633-7
An Artificial Metalloenzyme Based on a Copper Heteroscorpionate Enables sp3 C-H Functionalization via Intramolecular Carbene Insertion	LLM	10.1021/jacs.2c03311
An artificial nickel chlorinase based on the biotin-streptavidin technology	LLM	10.1039/D3CC05847F
Anion-π Enzymes	LLM	10.1021/acscentsci.6b00097
Anti-Markovnikov alkene oxidation by metal-oxo-mediated enzyme catalysis	LLM	10.1126/science.aao1482
Artificial Metalloenzymes based on TetR Proteins and Cu(II) for Enantioselective Friedel-Crafts Alkylation Reactions	LLM	10.1002/cctc.202000245
Biocatalytic Generation of Trifluoromethyl Radicals by Nonheme Iron Enzymes for Enantioselective Alkene Difunctionalization	LLM	10.1021/jacs.4c14310
Chemo- and Regioselective Dihydroxylation of Benzene to Hydroquinone Enabled by Engineered Cytochrome P450 Monooxygenase	LLM	10.1002/anie.201812093
Chemoenzymatic Platform for Synthesis of Chiral Organofluorines Based on Type II Aldolases	LLM	10.1002/anie.201906805

Chemoenzymatic Total Synthesis of Deoxy- epi- and Podophyllotoxin and a Biocatalytic Kinetic Resolution of Dibenzylbutyrolactones	LLM	10.1002/anie.201900926
Cofactor Binding Dynamics Influence the Catalytic Activity and Selectivity of an Artificial Metalloenzyme	LLM	10.1021/acscatal.0c01619
Computational Stabilization of a Non-Heme Iron Enzyme Enables Efficient Evolution of New Function	LLM	10.1002/anie.202414705
Controlling Non-Native Cobalamin Reactivity and Catalysis in the Transcription Factor CarH	LLM	10.1021/acscatal.1c04748
Directed Evolution of Artificial Metalloenzymes in Whole Cells	LLM	10.1002/anie.202110519
Directed evolution of Escherichia coli surface-displayed Vitreoscilla hemoglobin as an artificial metalloenzyme for the synthesis of 5-imino-1 2 4-thiadiazoles	LLM	10.1039/D4SC00005F
Discovery of a regioselectivity switch in nitrating P450s guided by molecular dynamics simulations and Markov models	LLM	10.1038/nchem.2474
Dual-Functional Small Molecules for Generating an Efficient Cytochrome P450BM3 Peroxygenase	LLM	10.1002/anie.201801592
Emergence of a catalytic tetrad during evolution of a highly active artificial aldolase	LLM	10.1038/nchem.2596
Enantiocomplementary Synthesis of γ-Nitroketones Using Designed and Evolved Carboligases	LLM	10.1021/jacs.6b11928
Enantiodivergent Radical Alkylation by Synergistic Lewis-Acid-Enzyme and Photoredox Catalysis	LLM	10.1002/anie.202500338
Enantioselective Aminohydroxylation of Styrenyl Olefins Catalyzed by an Engineered Hemoprotein	LLM	10.1002/anie.201812968
Enantioselective intermolecular benzylic C-H amination catalysed by an engineered iron-haem enzyme	LLM	10.1038/nchem.2783
Enantioselective Single and Dual α-C-H Bond Functionalization of Cyclic Amines via Enzymatic Carbene Transfer	LLM	10.1021/jacs.2c10775
Enantioselective Synthesis of Chiral Amines via Biocatalytic Carbene N-H Insertion	LLM	10.1021/acscatal.0c02794
Engineered Enzymes Enable Selective N-Alkylation of Pyrazoles With Simple Haloalkanes	LLM	10.1002/anie.202014239
Engineered P450 Atom-Transfer Radical Cyclases are Bifunctional Biocatalysts Reaction Mechanism and Origin of Enantioselectivity	LLM	10.1021/jacs.2c04937

Engineering transketolase to accept both unnatural donor and acceptor substrates and produce α-hydroxyketones	LLM	10.1111/febs.15108
Enhanced Sequence-Activity Mapping and Evolution of Artificial Metalloenzymes by Active Learning	LLM	10.1021/acscentsci.4c00258
Enzymatic construction of highly strained carbocycles	LLM	10.1126/science.aar4239
Expanding the Reactivity of Flavin Dependent Halogenases Toward Olefins via Enantioselective Intramolecular Haloetherification and Chemoenzymatic Oxidative Rearrangements	LLM	10.1021/acscatal.2c03383
First and second sphere interactions accelerate non-native N-alkylation catalysis by the thermostable, methanol-tolerant B12-dependent enzyme MtaC	LLM	10.1039/d3cc01071f
Flavin-dependent halogenases catalyze enantioselective olefin halocyclization	LLM	10.1038/s41467-021-23503-3
Hemoprotein-Catalyzed Cyclopropanation En Route to the Chiral Cyclopropanol Fragment of Grazoprevir	LLM	10.1002/cbic.201800652
Highly Stereoselective Synthesis of Fused Cyclopropane-γ-Lactams via Biocatalytic Iron-Catalyzed Intramolecular Cyclopropanation	LLM	10.1021/acscatal.9b05383
Improved Synthesis of 4-Cyanotryptophan and Other Tryptophan Analogues in Aqueous Solvent Using Variants of TrpB from Thermotoga maritima	LLM	10.1021/acs.joc.8b00517
Improving the Catalytic Performance of an Artificial Metalloenzyme by Computational Design	LLM	10.1021/jacs.5b06622
Insights into an efficient light-driven hybrid P450 BM3 enzyme from crystallographic spectroscopic and biochemical studies	LLM	10.1016/j.bbapap.2016.09.005
Light-driven reductive cyclization catalyzed by vitamin B12-based artificial photoenzymes	LLM	10.1039/d5ob00717h
Machine learning-assisted directed protein evolution with combinatorial libraries	LLM	10.1073/pnas.1901979116
NHC-Mediated Radical Acylation Catalyzed by Thiamine- and Flavin-Dependent Enzymes	LLM	10.1021/jacs.5c04484
Non-native Intramolecular Radical Cyclization Catalyzed by a B12 -Dependent Enzyme	LLM	10.1002/anie.202312893
P450-Catalyzed Regio- and Stereoselective Oxidative Hydroxylation of 6-Iodotetralone Preparative-Scale Synthesis of a Key Intermediate for Pd-Catalyzed Transformations	LLM	10.1021/acs.joc.7b02878

Photobiocatalytic Enantioselective Benzylic C(sp^3)-H Acylation Enabled by Thiamine-Dependent Enzymes via Intermolecular Hydrogen Atom Transfer	LLM	10.1021/jacs.5c01642
Photoenzymatic enantioselective intermolecular radical hydroalkylation	LLM	10.1038/s41586-020-2406-6
Photoenzymatic Enantioselective Intermolecular Radical Hydroamination	LLM	10.1038/s41929-023-00994-5
Production of Biobased Ethylbenzene by Cascade Biocatalysis with an Engineered Photodecarboxylase	LLM	10.1002/anie.202314566
Saturation Mutagenesis for Phenylalanine Ammonia Lyases of Enhanced Catalytic Properties	LLM	10.3390/biom10060838
Stereospecific radical coupling with a non-natural photodecarboxylase	LLM	10.1038/s41586-024-08004-9
Unlocking the function promiscuity of old yellow enzyme to catalyze asymmetric Morita-Baylis-Hillman reaction	LLM	10.1038/s41467-024-50141-2
Electricity-driven enzymatic dynamic kinetic oxidation	LLM	10.1038/s41586-025-09178-6
Emergence of a distinct mechanism of C-N bond formation in photoenzymes	LLM	10.1038/s41586-024-08138-w
Enantioselective Hydroxylation of Benzylic C(sp^3)-H Bonds by an Artificial Iron Hydroxylase Based on the Biotin-Streptavidin Technology	LLM	10.1021/jacs.0c02788
Enantioselective Synthesis of α-Trifluoromethyl Amines via Biocatalytic N-H Bond Insertion with Acceptor-Acceptor Carbene Donors	LLM	10.1021/jacs.1c10750
Engineering an Oxygen-Binding Protein for Photocatalytic CO_2 Reductions in Water	LLM	10.1002/anie.202215719
Engineering Chemoselectivity in Hemoprotein-Catalyzed Indole Amidation	LLM	10.1021/acscatal.9b02508
Enzymatic assembly of carbon-carbon bonds via iron-catalysed sp^3 C-H functionalization	LLM	10.1038/s41586-018-0808-5
Fast Knoevenagel Condensations Catalyzed by an Artificial Schiff-Base-Forming Enzyme	LLM	10.1021/jacs.6b00816
Gram-Scale Synthesis of Chiral Cyclopropane-Containing Drugs and Drug Precursors with Engineered Myoglobin Catalysts Featuring Complementary Stereoselectivity	LLM	10.1002/anie.201608680
Ground-State Electron Transfer as an Initiation Mechanism for Biocatalytic C-C Bond Forming Reactions	LLM	10.1021/jacs.1c04334

Highly Diastereo- and Enantioselective Synthesis of Nitrile-Substituted Cyclopropanes by Myoglobin-Mediated Carbene Transfer Catalysis	LLM	10.1002/anie.201810059
Highly diastereoselective and enantioselective olefin cyclopropanation using engineered myoglobin-based catalysts	LLM	10.1002/anie.201409928
E. coli surface display of streptavidin for directed evolution of an allylic deallylase	LLM	10.1039/c8sc00484f
Library design and screening protocol for artificial metalloenzymes based on the biotin-streptavidin technology	LLM	10.1038/nprot.2016.019
Myoglobin-Catalyzed Olefination of Aldehydes	LLM	10.1002/anie.201508817
Origin of high stereocontrol in olefin cyclopropanation catalyzed by an engineered carbene transferase	LLM	10.1021/acscatal.8b04073
Orthogonal Expression of an Artificial Metalloenzyme for Abiotic Catalysis	LLM	10.1002/cbic.201700397
Overriding Traditional Electronic Effects in Biocatalytic Baeyer-Villiger Reactions by Directed Evolution	LLM	10.1021/jacs.8b04742
Positioning-Group-Enabled Biocatalytic Oxidative Dearomatization	LLM	10.1021/acscentsci.9b00163
Promiscuity Guided Evolution of Decarboxylative Aldolases for Synthesis of Tertiary γ-Hydroxy Amino Acids	LLM	10.1002/anie.202422109
Repurposing Visible-Light-Excited Ene-Reductases for Diastereo- and Enantioselective Lactones Synthesis	LLM	10.1002/anie.202402673
Selective C-H Halogenation of Alkenes and Alkynes Using Flavin-Dependent Halogenases	LLM	10.1002/anie.202317860
Selective Functionalization of Aliphatic Amines via Myoglobin-catalyzed Carbene N-H Insertion	LLM	10.1055/s-0039-1690007
Stereodivergent Intramolecular Cyclopropanation Enabled by Engineered Carbene Transferases	LLM	10.1021/jacs.9b02700
Stereoselective amino acid synthesis by synergistic photoredox-pyridoxal radical biocatalysis	LLM	10.1126/science.adg2420
Synthesis of β-Quaternary Lactams Using Photoenzymatic Catalysis	LLM	10.1002/ajoc.202300274
Synthesis of β-Branched Tryptophan Analogues Using an Engineered Subunit of Tryptophan	LLM	10.1021/jacs.6b04836
Unlocking Asymmetric Michael Additions in an Archetypical Class I Aldolase by Directed Evolution	LLM	10.1021/acscatal.1c03911

Unlocking Reactivity of TrpB: A General Biocatalytic Platform for Synthesis of Tryptophan Analogues	LLM	10.1021/jacs.7b05007

S. Table 2. Curated and LLM extracted values:

Experiment name in EnzEngDB	# proteins	# rows	# fitness values	# reactions
An Enzymatic Platform for Primary Amination of 1-Aryl-2-alkyl Alkynes	9	28	22	19
An enzymatic platform for the asymmetric amination of primary, secondary and tertiary C(sp3)-H bonds	11	43	41	30
Asymmetric Alkylation of Ketones Catalyzed by Engineered TrpB	12	28	26	6
Biocatalytic Carbene Transfer Using Diazirines	6	14	12	9
Biocatalytic Construction of Chiral Pyrrolidines and Indolines via Intramolecular C(sp3)-H Amination	12	37	25	25
Biocatalytic One-Carbon Ring Expansion of Aziridines to Azetidines via a Highly Enantioselective [1,2]-Stevens Rearrangement	11	18	18	8
Biocatalytic Synthesis of α-Amino Esters via Nitrene C-H Insertion	51	97	54	43
Biocatalytic, Intermolecular C-H Bond Functionalization for the Synthesis of Enantioenriched Amides	13	56	38	20
Biocatalytic, Stereoconvergent Alkylation of (Z/E)-Trisubstituted Silyl Enol Ethers	6	21	19	14
Biocatalytic, Enantioenriched Primary Amination of Tertiary C-H Bonds	8	22	21	12
Chemodivergent C(sp3)-H and C(sp2)-H Cyanomethylation Using Engineered Carbene Transferases	3	18	17	18
Direct Enzymatic Synthesis of a Deep-Blue Fluorescent Noncanonical Amino Acid from Azulene and Serine	5	9	8	2
Directed Evolution of a Cytochrome P450 Carbene Transferase for Selective Functionalization of Cyclic Compounds	21	38	35	15
Directed evolution of cytochrome c for carbon-silicon bond formation: Bringing silicon to life	8	31	28	21
Directed evolution of enzymatic silicon-carbon bond cleavage in siloxanes	10	12	10	3
Diversity-Oriented Enzymatic Synthesis of Cyclopropane Building Blocks	11	13	10	2

Dual-Function Enzyme Catalysis for Enantioselective Carbon-Nitrogen Bond Formation	15	38	37	17
Enantio- and Diastereoenriched Enzymatic Synthesis of 1,2,3-Polysubstituted Cyclopropanes from (Z/ E)-Trisubstituted Enol Acetates	5	84	42	52
Enantiodivergent α-Amino C-H Fluoroalkylation Catalyzed by Engineered Cytochrome P450s	6	32	26	26
Engineered Cytochrome c-Catalyzed Lactone-Carbene B-H Insertion	5	13	11	9
Engineering Cytochrome P450s for EnantioselectiveCyclopropenation of Internal Alkynes	15	41	37	16
Enzymatic Assembly of Diverse Lactone Structures: An Intramolecular C-H Functionalization Strategy	26	50	30	24
Enzymatic Lactone-Carbene C–H Insertion to Build Contiguous Chiral Centers	10	200	96	16
Enzymatic Nitrogen Incorporation Using Hydroxylamine	5	20	20	10
Enzymatic Nitrogen Insertion into Unactivated C-H Bonds	23	81	35	61
Enzymatic Primary Amination of Benzylic and Allylic C(sp3)-H Bonds	9	54	49	41
Expanding Biocatalysis for Organosilane Functionalization: Enantioselective Nitrene Transfer to Benzylic Si–C–H Bonds	8	17	16	9
Genetically programmed chiral organoborane synthesis	9	20	20	16
Nitrene Transfer Catalyzed by a Non-Heme Iron Enzyme and Enhanced by Non-Native Small-Molecule Ligands	14	31	27	2
Nitroalkanes as Versatile Nucleophiles for Enzymatic Synthesis of Noncanonical Amino Acids	7	27	16	9
Protoglobin-Catalyzed Formation of cis-Trifluoromethyl-Substituted Cyclopropanes by Carbene Transfer	1	10	10	10
Reversing the Enantioselectivity of Enzymatic Carbene N-H Insertion Through Mechanism-Guided Protein Engineering	11	19	18	9
Selective Enzymatic Oxidation of Silanes to Silanols	4	19	18	12
Stereospecific Enzymatic Conversion of Boronic Acids to Amines	3	31	28	26
Tailoring Tryptophan Synthase TrpB for Selective Quaternary Carbon Bond Formation	7	27	19	10

LevSeq: Rapid Generation of Sequence-Function Data for Directed Evolution and Machine Learning (cis)	187	376	376	1
LevSeq: Rapid Generation of Sequence-Function Data for Directed Evolution and Machine Learning (trans)	202	472	472	1
Active learning-assisted directed evolution (cis)	396	397	396	1
Active learning-assisted directed evolution (trans)	396	397	397	1
Substrate-Aware Zero-Shot Predictors for Non-Native Enzyme Activities (ParLQ-h)	91	95	95	1
Substrate-Aware Zero-Shot Predictors for Non-Native Enzyme Activities (ParLQ-b)	91	95	94	1
Substrate-Aware Zero-Shot Predictors for Non-Native Enzyme Activities (ParLQ-f)	91	95	95	1
Substrate-Aware Zero-Shot Predictors for Non-Native Enzyme Activities (ParLQ-a)	490	493	488	1
Substrate-Aware Zero-Shot Predictors for Non-Native Enzyme Activities (ParLQ-e)	91	95	94	1
Substrate-Aware Zero-Shot Predictors for Non-Native Enzyme Activities (ParLQ-d)	91	95	95	1
Substrate-Aware Zero-Shot Predictors for Non-Native Enzyme Activities (ParLQ-g)	91	95	95	1
Substrate-Aware Zero-Shot Predictors for Non-Native Enzyme Activities (ParLQ-c)	91	95	95	1
Substrate-Aware Zero-Shot Predictors for Non-Native Enzyme Activities (ParLQ-i)	91	95	95	1
Machine learning-guided co-optimization of fitness and diversity facilitates combinatorial library design in enzyme engineering (Rma-CB)	151	162	74	1
Machine learning-guided co-optimization of fitness and diversity facilitates combinatorial library design in enzyme engineering (Rma-Csi)	151	162	41	1
Substrate-Aware Zero-Shot Predictors for Non-Native Enzyme Activities (PfTrpB_7-methyl)	241	242	242	1
Substrate-Aware Zero-Shot Predictors for Non-Native Enzyme Activities (PfTrpB_5-iodo)	241	242	242	1
Substrate-Aware Zero-Shot Predictors for Non-Native Enzyme Activities (PfTrpB_5-chloro)	241	242	241	1
Substrate-Aware Zero-Shot Predictors for Non-Native Enzyme Activities (PfTrpB_4-bromo)	241	242	237	1
Substrate-Aware Zero-Shot Predictors for Non-Native Enzyme Activities (PfTrpB_4-cyano)	241	242	242	1
Substrate-Aware Zero-Shot Predictors for Non-Native Enzyme Activities (PfTrpB_6-chloro)	241	242	241	1

Substrate-Aware Zero-Shot Predictors for Non-Native Enzyme Activities (PfTrpB_7-bromo)	68	69	69	1
Substrate-Aware Zero-Shot Predictors for Non-Native Enzyme Activities (PfTrpB_7-iodo)	241	242	240	1
Substrate-Aware Zero-Shot Predictors for Non-Native Enzyme Activities (PfTrpB_5-bromo)	241	242	238	1
Substrate-Aware Zero-Shot Predictors for Non-Native Enzyme Activities (PfTrpB_5-cyano)	241	242	240	1
The _-subunit of tryptophan synthase is a latent tyrosine synthase	10	26	25	5
**Totals EnzEngDB gold standard**	**1848**	**6777**	**6184**	**651**
A cell-penetrating artificial metalloenzyme regulates a gene switch in a designer mammalian cell	18	22	8	1
A designer enzyme for hydrazone and oxime formation featuring an unnatural catalytic aniline residue	3	3	3	1
A Diverse Library of Chiral Cyclopropane Scaffolds via Chemoenzymatic Assembly and Diversification of Cyclopropyl Ketones	4	34	26	30
A Photoenzymatic Strategy for Radical-Mediated Stereoselective Hydroalkylation with Diazo Compounds	7	22	19	15
Accessing non-natural reactivity by irradiating nicotinamide-dependent enzymes with light	3	12	11	10
An Artificial Heme Enzyme for Cyclopropanation Reactions	6	9	9	3
An Enzymatic Platform for Aniline Synthesis Through Oxidative Amination	7	37	29	30
An Enzymatic Platform for Asymmetric Synthesis of Si-Stereogenic Silanols	7	32	31	16
An Enzymatic Platform for the Highly Enantioselective and Stereodivergent Construction of Cyclopropyl-δ-lactones	10	42	22	20
Biocatalytic asymmetric aldol addition into unactivated ketones	1	31	29	13
Biocatalytic Strategy for Highly Diastereo- and Enantioselective Synthesis of 2 3-Dihydrobenzofuran-Based Tricyclic Scaffolds	4	21	16	8
Biocatalytic Strategy for the Highly Stereoselective Synthesis of CHF sub 2 sub -Containing Trisubstituted Cyclopropanes	9	35	32	19
Biocatalytic Synthesis of Allylic and Allenyl Sulfides through a Myoglobin-Catalyzed Doyle-Kirmse Reaction	13	29	25	17

Catalytic iron-carbene intermediate revealed in a cytochrome c carbene transferase	1	1	1	1
Catalytic promiscuity enabled by photoredox catalysis in nicotinamide-dependent oxidoreductases	4	18	17	13
Chemoselective Cyclopropanation over Carbene Y-H Insertion Catalyzed by an Engineered Carbene Transferase	6	20	18	7
Dehaloperoxidase Catalyzed Stereoselective Synthesis of Cyclopropanol Esters	9	30	18	17
Design and Evolution of an Artificial Friedel-Crafts Alkylation Enzyme Featuring an Organoboronic Acid Residue	10	61	36	37
Design and Evolution of an Enzyme for the Asymmetric Michael Addition of Cyclic Ketones to Nitroolefins by Enamine Catalysis	12	30	25	16
Directed Evolution of a Fluorinase for Improved Fluorination Efficiency with a Non-native Substrate	4	4	4	1
Directed Evolution of an Artificial Imine Reductase	7	7	4	1
Diverse Engineered Heme Proteins Enable Stereodivergent Cyclopropanation of Unactivated Alkenes	6	20	18	13
A De Novo Metalloenzyme for Cerium Photoredox Catalysis	2	3	3	1
Abiotic reduction of ketones with silanes catalysed by carbonic anhydrase through an enzymatic zinc hydride	1	29	16	28
An Artificial Metalloenzyme Based on a Copper Heteroscorpionate Enables sp3 C-H Functionalization via Intramolecular Carbene Insertion	21	29	24	6
An artificial nickel chlorinase based on the biotin-streptavidin technology	55	56	34	1
Anion-π Enzymes	18	30	23	1
Anti-Markovnikov alkene oxidation by metal-oxo-mediated enzyme catalysis	9	9	9	1
Artificial Metalloenzymes based on TetR Proteins and Cu(II) for Enantioselective Friedel-Crafts Alkylation Reactions	3	23	12	8
Biocatalytic Generation of Trifluoromethyl Radicals by Nonheme Iron Enzymes for Enantioselective Alkene Difunctionalization	10	31	20	20
Chemo- and Regioselective Dihydroxylation of Benzene to Hydroquinone Enabled by Engineered Cytochrome P450 Monooxygenase	7	7	7	1

Chemoenzymatic Platform for Synthesis of Chiral Organofluorines Based on Type II Aldolases	4	8	5	3
Chemoenzymatic Total Synthesis of Deoxy- epi- and Podophyllotoxin and a Biocatalytic Kinetic Resolution of Dibenzylbutyrolactones	1	13	11	1
Cofactor Binding Dynamics Influence the Catalytic Activity and Selectivity of an Artificial Metalloenzyme	13	17	15	3
Computational Stabilization of a Non-Heme Iron Enzyme Enables Efficient Evolution of New Function	8	8	7	2
Controlling Non-Native Cobalamin Reactivity and Catalysis in the Transcription Factor CarH	11	19	18	9
Directed Evolution of Artificial Metalloenzymes in Whole Cells	7	20	20	14
Directed evolution of Escherichia coli surface-displayed Vitreoscilla hemoglobin as an artificial metalloenzyme for the synthesis of 5-imino-1 2 4-thiadiazoles	3	34	22	32
Discovery of a regioselectivity switch in nitrating P450s guided by molecular dynamics simulations and Markov models	4	4	4	1
Dual-Functional Small Molecules for Generating an Efficient Cytochrome P450BM3 Peroxygenase	7	7	7	1
Emergence of a catalytic tetrad during evolution of a highly active artificial aldolase	2	10	8	9
Enantiocomplementary Synthesis of γ-Nitroketones Using Designed and Evolved Carboligases	3	21	10	6
Enantiodivergent Radical Alkylation by Synergistic Lewis-Acid-Enzyme and Photoredox Catalysis	47	92	48	21
Enantioselective Aminohydroxylation of Styrenyl Olefins Catalyzed by an Engineered Hemoprotein	8	21	19	13
Enantioselective intermolecular benzylic C-H amination catalysed by an engineered iron-haem enzyme	5	32	28	17
Enantioselective Single and Dual α-C-H Bond Functionalization of Cyclic Amines via Enzymatic Carbene Transfer	13	32	26	13
Enantioselective Synthesis of Chiral Amines via Biocatalytic Carbene N-H Insertion	14	57	37	27
Engineered Enzymes Enable Selective N-Alkylation of Pyrazoles With Simple Haloalkanes	37	59	40	13
Engineered P450 Atom-Transfer Radical Cyclases are Bifunctional Biocatalysts Reaction Mechanism and Origin of Enantioselectivity	8	8	7	1

Engineering transketolase to accept both unnatural donor and acceptor substrates and produce _-hydroxyketones	5	7	6	6
Enhanced Sequence-Activity Mapping and Evolution of Artificial Metalloenzymes by Active Learning	8	8	8	1
Enzymatic construction of highly strained carbocycles	17	62	36	27
Expanding the Reactivity of Flavin Dependent Halogenases Toward Olefins via Enantioselective Intramolecular Haloetherification and Chemoenzymatic Oxidative Rearrangements	6	20	17	14
First and second sphere interactions accelerate non-native N-alkylation catalysis by the thermostable, methanol-tolerant B12-dependent enzyme MtaC	4	19	15	15
Flavin-dependent halogenases catalyze enantioselective olefin halocyclization	7	16	8	4
Hemoprotein-Catalyzed Cyclopropanation En Route to the Chiral Cyclopropanol Fragment of Grazoprevir	14	15	15	1
Highly Stereoselective Synthesis of Fused Cyclopropane-γ-Lactams via Biocatalytic Iron-Catalyzed Intramolecular Cyclopropanation	6	11	11	5
Improved Synthesis of 4-Cyanotryptophan and Other Tryptophan Analogues in Aqueous Solvent Using Variants of TrpB from Thermotoga maritima	3	18	18	6
Improving the Catalytic Performance of an Artificial Metalloenzyme by Computational Design	9	15	14	4
Insights into an efficient light-driven hybrid P450 BM3 enzyme from crystallographic spectroscopic and biochemical studies	3	3	3	1
Light-driven reductive cyclization catalyzed by vitamin B12-based artificial photoenzymes	9	18	16	9
Machine learning-assisted directed protein evolution with combinatorial libraries	9	9	8	1
NHC-Mediated Radical Acylation Catalyzed by Thiamine- and Flavin-Dependent Enzymes	16	29	28	13
Non-native Intramolecular Radical Cyclization Catalyzed by a B12 -Dependent Enzyme	6	18	12	3
P450-Catalyzed Regio- and Stereoselective Oxidative Hydroxylation of 6-Iodotetralone Preparative-Scale Synthesis of a Key Intermediate for Pd-Catalyzed Transformations	8	8	8	1
Photobiocatalytic Enantioselective Benzylic C(sp3)-H Acylation Enabled by Thiamine-Dependent Enzymes via Intermolecular Hydrogen Atom Transfer	8	60	39	26

Photoenzymatic enantioselective intermolecular radical hydroalkylation	9	25	18	16
Photoenzymatic Enantioselective Intermolecular Radical Hydroamination	53	73	52	21
Production of Biobased Ethylbenzene by Cascade Biocatalysis with an Engineered Photodecarboxylase	9	9	9	1
Saturation Mutagenesis for Phenylalanine Ammonia Lyases of Enhanced Catalytic Properties	6	6	5	1
Stereospecific radical coupling with a non-natural photodecarboxylase	17	44	31	21
Unlocking the function promiscuity of old yellow enzyme to catalyze asymmetric Morita-Baylis-Hillman reaction	6	20	15	6
Electricity-driven enzymatic dynamic kinetic oxidation	28	81	37	26
Emergence of a distinct mechanism of C-N bond formation in photoenzymes	6	29	26	24
Enantioselective Hydroxylation of Benzylic C(sp3)-H Bonds by an Artificial Iron Hydroxylase Based on the Biotin-Streptavidin Technology	31	62	19	1
Enantioselective Synthesis of α-Trifluoromethyl Amines via Biocatalytic N-H Bond Insertion with Acceptor-Acceptor Carbene Donors	5	29	26	25
Engineering an Oxygen-Binding Protein for Photocatalytic CO2 Reductions in Water	6	12	10	1
Engineering Chemoselectivity in Hemoprotein-Catalyzed Indole Amidation	7	17	17	8
Enzymatic assembly of carbon-carbon bonds via iron-catalysed sp3 C-H functionalization	9	45	42	30
Fast Knoevenagel Condensations Catalyzed by an Artificial Schiff-Base-Forming Enzyme	2	8	8	4
Gram-Scale Synthesis of Chiral Cyclopropane-Containing Drugs and Drug Precursors with Engineered Myoglobin Catalysts Featuring Complementary Stereoselectivity	16	16	14	1
Ground-State Electron Transfer as an Initiation Mechanism for Biocatalytic C-C Bond Forming Reactions	5	47	37	36
Highly Diastereo- and Enantioselective Synthesis of Nitrile-Substituted Cyclopropanes by Myoglobin-Mediated Carbene Transfer Catalysis	2	22	21	21
Highly diastereoselective and enantioselective olefin cyclopropanation using engineered myoglobin-based catalysts	10	18	17	9

E. coli surface display of streptavidin for directed evolution of an allylic deallylase	7	7	7	1
Library design and screening protocol for artificial metalloenzymes based on the biotin-streptavidin technology	1	1	1	1
Myoglobin-Catalyzed Olefination of Aldehydes	10	40	37	17
Origin of high stereocontrol in olefin cyclopropanation catalyzed by an engineered carbene transferase	3	7	5	5
Orthogonal Expression of an Artificial Metalloenzyme for Abiotic Catalysis	6	18	12	3
Overriding Traditional Electronic Effects in Biocatalytic Baeyer-Villiger Reactions by Directed Evolution	12	19	8	1
Positioning-Group-Enabled Biocatalytic Oxidative Dearomatization	1	13	13	13
Promiscuity Guided Evolution of Decarboxylative Aldolases for Synthesis of Tertiary γ-Hydroxy Amino Acids	5	17	16	7
Repurposing Visible-Light-Excited Ene-Reductases for Diastereo- and Enantioselective Lactones Synthesis	4	17	16	15
Selective C-H Halogenation of Alkenes and Alkynes Using Flavin-Dependent Halogenases	1	15	12	15
Selective Functionalization of Aliphatic Amines via Myoglobin-catalyzed Carbene N-H Insertion	12	30	21	17
Stereodivergent Intramolecular Cyclopropanation Enabled by Engineered Carbene Transferases	9	34	26	14
Stereoselective amino acid synthesis by synergistic photoredox-pyridoxal radical biocatalysis	14	47	37	21
Synthesis of β-Quaternary Lactams Using Photoenzymatic Catalysis	2	9	9	8
Synthesis of β-Branched Tryptophan Analogues Using an Engineered Subunit of Tryptophan Synthase	3	3	3	1
Unlocking Asymmetric Michael Additions in an Archetypical Class I Aldolase by Directed Evolution	8	22	12	11
Unlocking Reactivity of TrpB: A General Biocatalytic Platform for Synthesis of Tryptophan Analogues	8	25	21	7
**Totals LLM**	**943**	**2422**	**1803**	**1088**

S. Table 3. In house and large curated datasets (each for a specific reaction):

Experiment name in EnzEngDB	# proteins	# rows	# fitness values

A combinatorially complete epistatic fitness landscape in an enzyme active site (TrpB_3H)	8930	8930	8930
Evaluation of Machine Learning-Assisted Directed Evolution Across Diverse Combinatorial Landscapes (DHFR)	9261	9261	9261
A combinatorially complete epistatic fitness landscape in an enzyme active site (TrpB_3A)	9230	9230	9225
A combinatorially complete epistatic fitness landscape in an enzyme active site (TrpB_3E)	9222	9222	9222
A combinatorially complete epistatic fitness landscape in an enzyme active site (TrpB_3B)	9257	9257	9254
A combinatorially complete epistatic fitness landscape in an enzyme active site (TrpB_3F)	8543	8543	8543
A combinatorially complete epistatic fitness landscape in an enzyme active site (TrpB_3G)	8709	8709	8709
A combinatorially complete epistatic fitness landscape in an enzyme active site (TrpB_3C)	9255	9255	9252
A combinatorially complete epistatic fitness landscape in an enzyme active site (TrpB_3D)	8968	8968	8968
Evaluation of Machine Learning-Assisted Directed Evolution Across Diverse Combinatorial Landscapes (T7)	6725	6725	429
A combinatorially complete epistatic fitness landscape in an enzyme active site (TrpB_B4)	193170	193170	193170
Evaluation of Machine Learning-Assisted Directed Evolution Across Diverse Combinatorial Landscapes (TEV)	159132	159132	158324
A combinatorially complete epistatic fitness landscape in an enzyme active site (TrpB_3I)	9018	9018	9018
**Totals**		**449420**	**442305**

SI 5: **Reactions from LLM Extraction and the Manually Curated Gold-Standard Dataset**

DEDB675 1.0

LLM

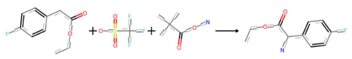

Manual

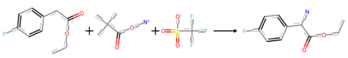

DEDB809 1.0

LLM

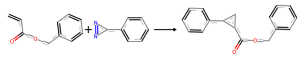

Manual

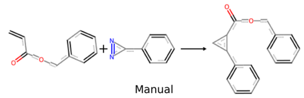

DEDB434 1.0

LLM

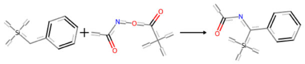

Manual

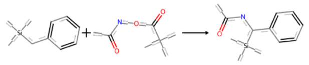

DEDB1217 1.0

LLM

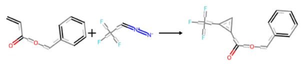

Manual

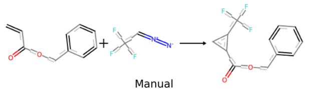

DEDB363 1.0

LLM

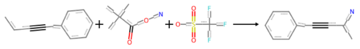

Manual

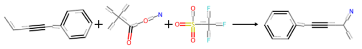

DEDB0 1.0

LLM

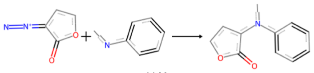

Manual

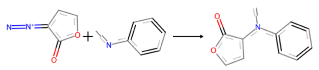

DEDB478 1.0

LLM

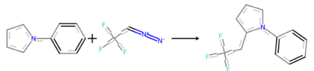

Manual

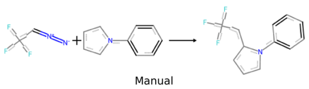

DEDB510 1.0

LLM

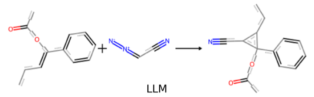

Manual

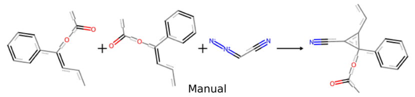

DEDB1290 1.0

LLM

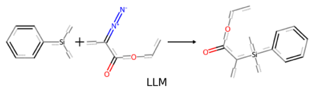

Manual

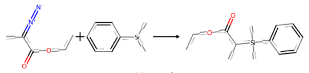

DEDB772 1.0

LLM

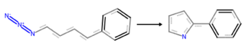

Manual

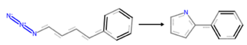

DEDB38 1.0

LLM

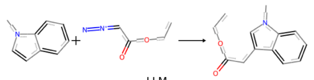

Manual

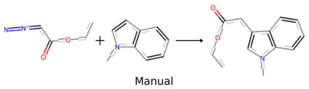

DEDB915 1.0

LLM

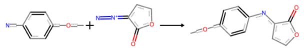

Manual

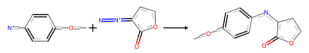

DEDB1072 1.0

LLM

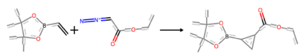

Manual

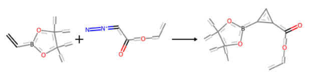

DEDB894 0.95

LLM

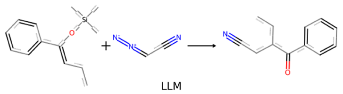

Manual

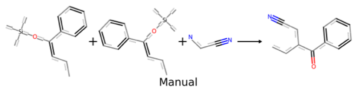

DEDB997 0.94

LLM

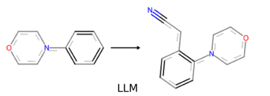

Manual

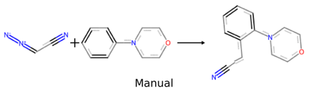

DEDB863 0.91

LLM

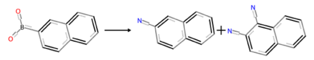

Manual

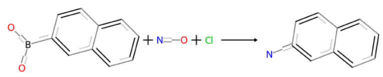

DEDB863 0.91

LLM

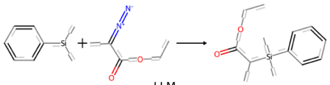

Manual

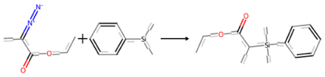

DEDB451 0.89

LLM

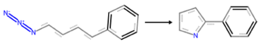

Manual

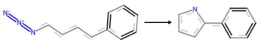

DEDB117 0.85

LLM

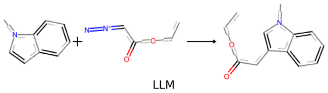

Manual

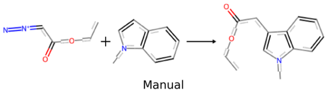

DEDB76 0.79

LLM

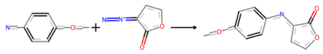

Manual

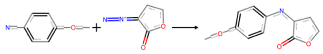

DEDB988 0.74

LLM

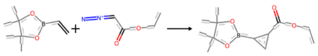

Manual

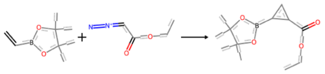

DEDB317 0.74

LLM

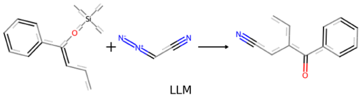

Manual

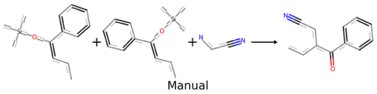

DEDB594 0.72

LLM

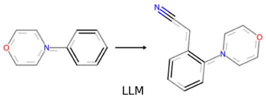

Manual

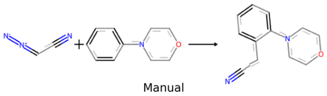

DEDB1085 0.72

LLM

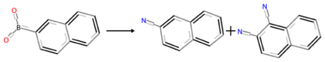

Manual

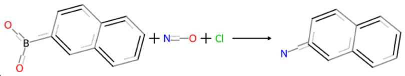

DEDB1161 0.71

LLM

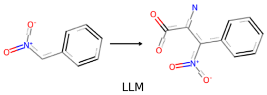

Manual

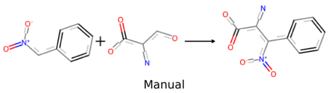

DEDB934 0.69

LLM

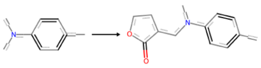

Manual

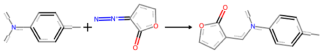

DEDB336 0.69

LLM

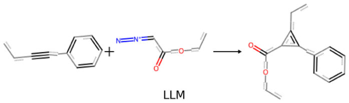

Manual

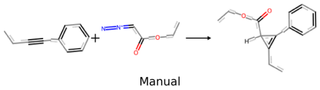

DEDB1239 0.66

LLM

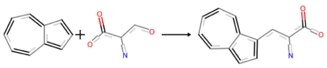

Manual

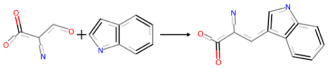

DEDB1227 0.57

LLM

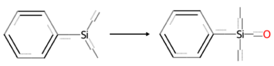

Manual

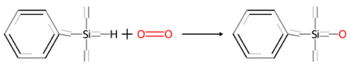

DEDB1031 0.51

LLM

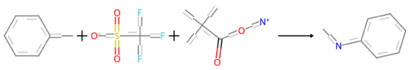

Manual

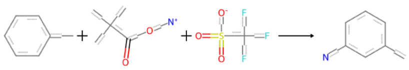

DEDB1059 0.37

LLM

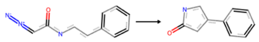

Manual

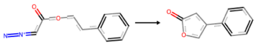

DEDB841 0.21

LLM

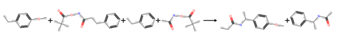

Manual

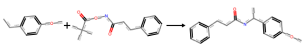

SI 6: **Evaluation of the LLM Extraction Pipeline Against the Gold-Standard Dataset**

To evaluate the LLM extraction pipeline, we computationally compared the sequences and reactions extracted by the LLM with those from the gold standard dataset. First, we assessed how many extracted papers contained both the original parent sequence and the corresponding reaction. For this, we used the first sequence and reaction from the manual extraction as the reference features. The corresponding LLM-extracted data was searched for reactions matching the manual reaction, selecting only the most similar row (i.e., the sequence corresponding to the most similar reaction). These rows were then filtered for missing amino_acid_substitutions (i.e., parent sequences), which were subsequently checked for sequence similarity using Hamming distance and Levenshtein distance, allowing for truncation and variable start positions (as it is common for researchers to start at the first index and omit the methionine). Any disagreements in residues or truncations were recorded. The most common truncation difference was omission of the His-tag, which was filtered out during postprocessing of the manually curated dataset.

This process resulted in 10 correct sequences, five single mutations (two of which were identified as errors in the manually curated data, and three attributable to the LLM), seven sequences deemed completely incorrect (Hamming distance > 100), and one missing sequence. We next checked whether the substitutions were part of the correct lineage or randomly introduced. We found that some positions were from the correct lineage, suggesting these errors were likely due to incorrect additions; however, this was inconsistent beyond the single substitutions. Therefore, we considered a case successful only in the first 15 of 32 instances (four cases were omitted as they could not be extracted by the LLM). Of these, eight had correct reactions (similarity score > 0.8), resulting in a 9 out of 27 paper success rate (33%) for the LLM extraction, which included two cases where the parent sequence was correctly identified by the LLM and improved the manual curation.

SI 7: **Automated and Manual Validation Steps for LLM-Extracted Papers**

Extraction Pipeline Sequence Validation

The sequence extractor checks whether a DNA sequence contains internal stop codons and rejects it if any are found. Additionally, up to five sequence queries are performed to generate consensus reads for the extracted sequences. The cleanup step also includes a sanity check, ensuring that a sequence is only populated if the identified mutations match those of the parent sequence. For example, if mutations V56Y and A90S are identified, the parent sequence must contain V56 and A90 for the child sequence to be populated. The same logic applies in reverse for child-to-parent sequence generation.

Extraction Pipeline Reaction Validation

The LLM extractor is specifically prompted to extract only the ID and map it to an existing IUPAC name in the file. If no IUPAC name is found, the extraction pipeline leaves the IUPAC section empty or marks it as "not mentioned in file."

Manual Validation

For each final CSV, the extractor first reviews the amino_acid_substitution column and verifies that each variant's substitution matches the paper's description. The extractor then manually copies and pastes the sequence from the file (if available) or retrieves it from the PDB if the notes field in enzyme_sequence_data.CSV indicates a PDB source. If the sequence matches and the mutation matches, the sequence extraction for the paper is deemed successful. This is because mutations are not propagated through the pipeline but are calculated only at the final formatting step. Therefore, if one sequence and its mutation are correct, all related sequences are also correct. In some cases, sequences are manually filled in, and the final formatting step is run manually to generate the final CSV.

For reaction validation, the extractor checks the manuscript and supplementary information (SI) for an IUPAC name section. If it exists, each substrate and product ID is manually verified to ensure correct mapping. If no IUPAC section exists, the extractor verifies that the name extracted in 3b_substrate_scope.CSV appears anywhere in the text; if not, the entire entry is removed. Since IUPAC names are programmatically converted to SMILES using NCI, PubChem, and OPSIN, accurate IUPAC extraction ensures correct SMILES strings. If all IUPAC names map correctly to their corresponding IDs, the reaction extraction is deemed successful. In rare cases, IUPAC names are manually added, and the final formatting step is run manually to produce the final CSV.
